# Cardiac thrombus detected by cardiac computed tomography angiography in patients with acute ischemic stroke: a meta-analysis

**DOI:** 10.3389/fneur.2024.1453683

**Published:** 2024-09-10

**Authors:** Buyun Xu, Ye Du, Zhangjie Yu, Yong Sun, Meixiang Xiang

**Affiliations:** ^1^State Key Laboratory of Transvascular Implantation Devices, Department of Cardiology, The Second Affiliated Hospital, Zhejiang University School of Medicine, Hangzhou, China; ^2^Department of Cardiology, Shaoxing People’s Hospital (Shaoxing Hospital of Zhejiang University), Shaoxing, China; ^3^Department of Neurology, Shaoxing People’s Hospital (Shaoxing Hospital of Zhejiang University), Shaoxing, China

**Keywords:** atrial fibrillation, cardiac computed tomography angiography, stroke, thrombus, cardioembolism

## Abstract

**Background:**

Detecting cardiac thrombus in patients with acute ischemic stroke is crucial in determine stroke etiology and predict prognosis. However, the prevalence of cardiac thrombus in patients with acute ischemic stroke is unclear.

**Object:**

This study aimed to evaluate the prevalence of cardiac thrombus detected by cardiac computed tomography angiography (CCTA) in patients with acute ischemic stroke through a meta-analysis.

**Methods:**

Embase, Web of Science, MEDLINE, and CENTRAL were searched from January 1, 2000, to May 1, 2024. We included observational studies enrolling patients who underwent CCTA within 1 month following acute ischemic stroke, and reporting the incidence of cardiac thrombi on CCTA. Meta-analysis was performed using random effects models.

**Results:**

Twenty-six studies involving 4,516 patients were identified. The pooled prevalence of cardiac thrombus detected on CCTA in patients with acute ischemic stroke was 0.08 (95% confidence interval [CI]: 0.06–0.11). Inter-study heterogeneity was high (I^2^ = 88%). Among stroke type, the prevalence of atrial fibrillation, timing of CCTA and CCTA technology, the prevalence of atrial fibrillation was the only factor associated with cardiac thrombi prevalence detected by CCTA. However, atrial fibrillation was not documented in 41.5% of the patients with cardiac thrombi.

**Conclusion:**

CCTA is a useful non-invasive imaging approach for detecting cardiac thrombus in patients with acute ischemic stroke, which might be helpful to determine the stroke etiology.

## 1 Introduction

Acute ischemic stroke (AIS) is a leading cause of morbidity and mortality worldwide. Identifying the etiology of stroke is crucial for preventing recurrence (1). However, in approximately one-third of AIS cases, the cause remains unknown after a systemic evaluation, classifying these as cryptogenic strokes (1). Cardioembolism might explain some cryptogenic strokes; these patients may require anticoagulant therapy instead of antiplatelet therapy. Evaluating cardiac thrombosis is essential for identifying the etiology of stroke (2). Furthermore, cardiac thrombus detected by imaging modality is a major contributor to poor prognosis in patients with cardioembolic stroke (3, 4). Therefore, detecting cardiac thrombosis is crucial in the acute stroke setting.

Traditionally, echocardiography (both transthoracic and transesophageal) has been the primary imaging modality for evaluating cardiac thrombosis. Although transthoracic echocardiography (TTE) is convenient, its sensitivity in detecting cardiac thrombosis, especially left atrial appendage (LAA) thrombosis, is low (5). Due to its proximity to the left atrial appendage, transesophageal echocardiography (TEE) is the standard method for evaluating thrombus in the left atrium (LA) and LAA (5). However, TEE is often performed days after the onset of AIS, typically following intravenous thrombolysis and anti-thrombosis treatment, which may reduce the likelihood of detecting cardiac thrombus. Moreover, TEE is a semi-invasive, time-consuming, and patient-unfriendly procedure.

Cardiac computed tomography angiography (CCTA) provides a non-invasive alternative, allowing for a detailed assessment of potential embolic sources, and can be performed in the hyperacute period (6, 7). However, the use of CCTA in the acute stroke setting remains controversial. This meta-analysis aimed to evaluate the prevalence of intracardiac thrombi detected by CCTA in patients with AIS and to offer insights into its integration into clinical protocols for the management of AIS.

## 2 Methods

The study was conducted and reported in accordance with the PRISMA (Preferred Reporting Items for Systematic Reviews and Meta-Analyses) (8). Detailed PRISMA reporting is shown in Supplementary Table S1.

### 2.1 Search strategy and eligibility criteria

We systematically searched Embase, Web of Science, MEDLINE, and CENTRAL for studies reported from January 1, 2000, to May 1, 2024, using various permutations of ischemic stroke and cardiac CT angiography. Supplementary Table S2 provides the detailed search strategy. No language restrictions were imposed. Additionally, we checked the reference lists of all the key articles for further eligible studies.

Studies were included if they met the following criteria: (1) enrolled patients with AIS (2), the patients underwent CCTA during the AIS admission, and (3) reported the incidence of cardiac thrombi on CCTA. Duplicate reports were excluded from analysis. For studies that contained overlapping populations, the largest study was considered for analysis. Abstracts of meeting proceedings were excluded unless full texts were published in a peer-reviewed journal. Eligible articles were selected independently by two investigators, with disparities resolved through discussion.

### 2.2 Data extraction and quality assessment

Two independent authors extracted following data from each eligible study: the year of publication, sample size, study design, patient characteristics, CT technology, interval between stroke and CCTA and incidence and the location of cardiac thrombus on CCTA.

Two independent reviewers evaluated the risk of bias in the included studies, utilizing the Joanna Briggs Institute Critical Appraisal Checklist for Studies Reporting Prevalence Data (9). This checklist is a nine-item tool where each question was rated as 1 for “Yes” and 0 for “No” or “Unclear.” Detailed information on this tool is provided in Supplementary Table S3. Studies with scores of 0–5, 6–7, and 8–9 were considered to have a high, medium, and low risk of bias, respectively. All results were cross-checked, and any disagreements were resolved through discussion.

### 2.3 Statistical analysis

Owing to the clinical heterogeneity of the included study populations, all analyses were conducted using random-effects models. Generalized linear mixed models were used to pool data across the studies, and cardiac thrombus prevalence was reported with 95% confidence intervals (CIs). Inter-study heterogeneity was assessed using Cochran’s Q test and expressed with the I^2^ statistic, with significant heterogeneity presumed when *p* < 0.05 and/or I^2^ > 50%.

Sensitivity analyses were performed to evaluate the robustness of the pooled prevalence estimates and examine the influence of individual studies on the pooled results and inter-study heterogeneity. Sensitivity analyses excluded studies with: (1) a medium or high risk of bias (score < 8), (2) a study sample with <100 patients, (3) studies conducted before 2020, and (4) retrospective studies. The *p*-values were determined by testing the homogeneity of cardiac thrombus prevalence detected by CCTA between the included and excluded studies. In addition, to evaluate the effect of individual studies on the results, sensitivity analyses were performed by removing each study one at a time.

Subgroup analyses were conducted to identify factors related to the prevalence of cardiac thrombus detected by CCTA. These subgroups were based on: (1) stroke type (embolic stroke/cardioembolic stroke vs. unclassified stroke/transient ischemic attack [TIA]), (2) prevalence of atrial fibrillation among the study population (≥30% vs. <30%), (3) time interval from symptom onset to CCTA (within 24 h vs. after 24 h), and (4) CT technology (single-phase vs. double-phase and ECG-gated vs. non-ECG-gated). The *p*-values for the subgroup analysis were determined by testing homogeneity. Publication bias was qualitatively assessed using a funnel plot.

A two-sided *p*-value of <0.05 was considered significant. Statistical analyses were performed using the meta/metafor package in R statistical software (Version 4.0.1, Vienna, Austria).

## 3 Results

### 3.1 Characteristics of studies and quality assessment

**Figure 1:**
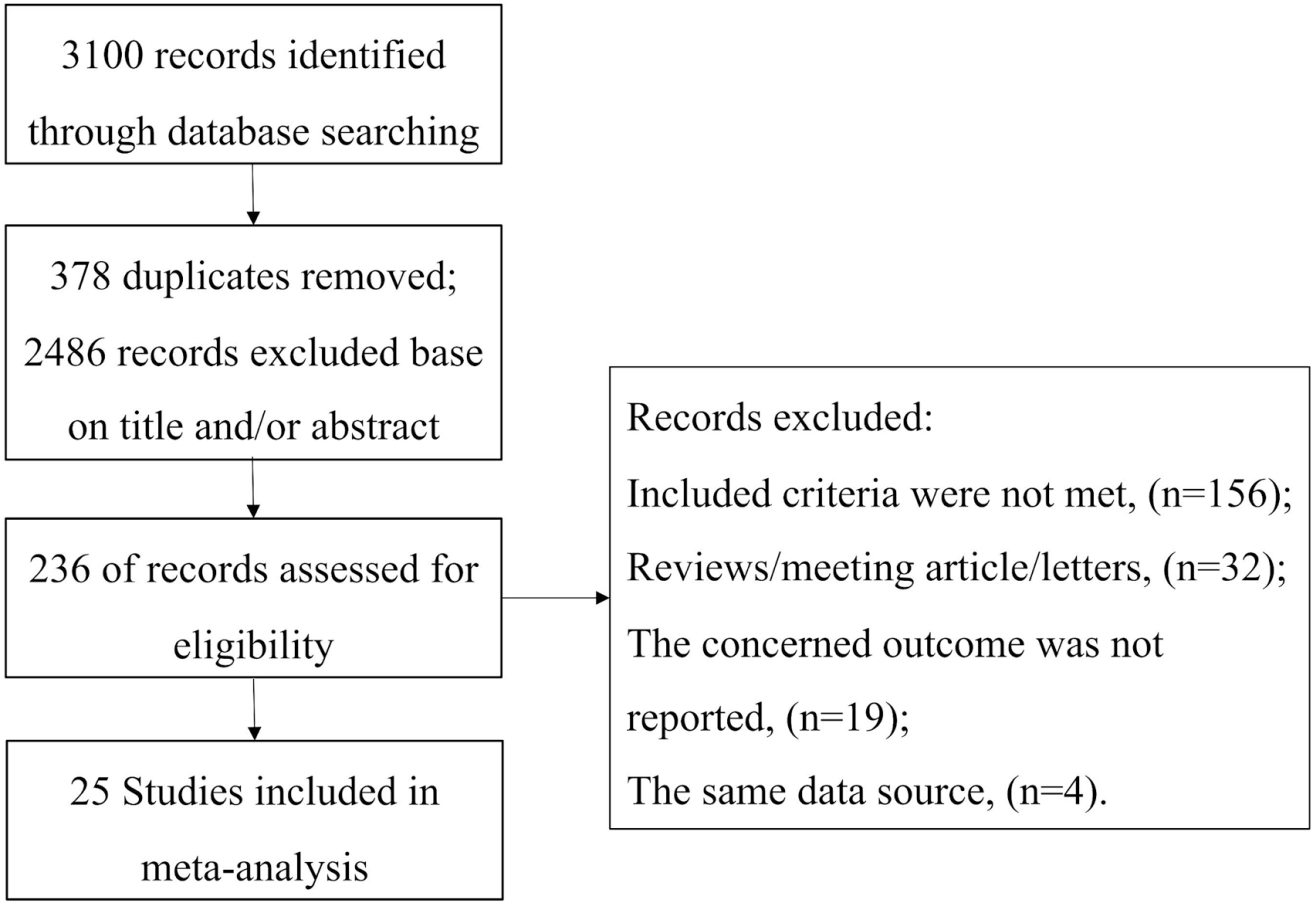
Flow diagram of the selection process of eligible articles.

The study selection process is illustrated in Figure 1. Initially, 3,100 references were identified through database searches. After screening the titles and abstracts, 2,864 articles were excluded. Subsequently, a total of 236 full-text articles were evaluated for eligibility. Ultimately, 25 studies involving 4,516 patients were included in the meta-analysis (2, 3, 6, 10–31).

The characteristics of the patients in the included studies are summarized in Table 1. All studies were single-center, and 16 studies employed a prospective design. Fourteen studies enrolled patients who had experienced a stroke or TIA, regardless of the stroke classification. The remaining 11 studies focused on patients with embolic stroke, with five studies specifically including patients classified as having cardioembolic stroke according to the TOAST classification. The time interval from stroke onset to CCTA scan was reported in 17 studies and CCTA was performed within 24 h in 10 studies. Dual-phase CCTA was used in 12 studies, and most studies used CT scanners with ≥64 slices.

**Table 1:** Characteristics of the included studies.

Study	N	Study type	Stroke type^a^	Age^b^	Male (%)	AF (%)	CT slice	CT technology	Timing of CCTA
Ajlan et al. (10)	47	Retrospective	CE	52 ± 11	53.2	4.2	128 s	Single-phase; ECG-gated	NR
Bernard et al. (11)	324	Retrospective	Stroke	With thrombus:82 ± 12; Without thrombus:74 ± 14	51.9	21.0	320 s	Single-phase; ECG-gated	Following emergency head CT
Philippe et al. (12)	415	Retrospective	Stroke	77 (60–86)	53.0	NR	320 s	Single-phase; ECG-gated	Following emergency head CT
Austein et al. (13)	60	Retrospective	CE	76 (72–82)	43.3	43.3	64 s	Two-phase; ECG-gated	Following emergency head CT
Kauw et al. (2)	353	Prospective	Stroke +TIA	67 ± 14	60.9	16.4	192 s	Single-phase; non-gated	Within 9 h
Holswilder et al. (14)	67	NR	Stroke +TIA	68 (51–89)	68.7	3.0	320 s	Single-phase; ECG-gated	Median interval: 2 days
Hur et al. (15)	55	Prospective	Stroke	With thrombus: 63 ± 10; Without thrombus:59 ± 14	65.5	60.0	64 s	Two-phase; ECG-gated	NR
Hur et al. (16)	137	Prospective	Stroke	61 ± 13	69.3	41.6	64 s	Two-phase; ECG-gated	Within 1 week after stroke
Hur et al. (17)	83	Prospective	Stroke^c^	63 ± 11	67.5	4.8	256 s	Single-phase; ECG-gated	NR
Lee et al. (18)	374	Prospective	Stroke	63 ± 13	67.9	12.0	128 s	Single-phase; ECG-gated	Within 1 week after stroke
Iwasaki et al. (19)	184	NR	ES	69 ± 13	66.3	14.1	64 s	Two-phase; ECG-gated	NR
Boussel et al. (20)	46	Prospective	Stroke	63 ± 11	82.6	6.5	40s	Two-phase; ECG-gated	NR
Yeo et al. (21)	20	Prospective	Stroke	64 ± 12	65.0	20.0	64 s	Single-phase; non-gated	Following emergency head CT
Rinkel et al. (22)	452	Prospective	Stroke	With thrombus: 76 (63–87); Without thrombus: 72 (62–80)	59.3	17.0	128 s	Single-phase; ECG-gated	Median interval: 32 min
Lee et al. (23)	120	Prospective	Stroke +TIA + SMS	73 (63–81)	55.0	26.7	64 s	Single-phase; non-gated	Following emergency head CT
Sipola et al. (24)	140	Prospective	CE	60 ± 10	67.9	NR	16 s and 64 s	Single-phase; ECG-gated	NR
Barnea et al. (25)	129	Retrospective	ESUS	74 ± 10	55.0	0.0%	256 s	Two-phase; ECG-gated	NR
Kawada et al. (26)	57	Prospective	ES	Median: 73; minmun:38; maximum: 93	68.4	NR	NR	Two-phase; ECG-gated	NR
Kim et al. (27)	314	Prospective	ES	65 ± 13	59.2	22.9	64 s	Two-phase; ECG-gated	Within 1 week after stroke
Ko et al. (28)	124	Prospective	ES	CCTA only: 76 (66–80); CCTA and TEE: 67 (58–72)	54.0	31.5	64 s	Two-phase; ECG-gated	Median interval: 2 days
Popkirov et al. (29)	21	Retrospective	ESUS+CE	CE: 78 ± 8; ESUS: 80 ± 3	52.4	81.0	NR	Single-phase; non-gated	Following emergency head CT
Senadeera et al. (30)	303	Retrospective	Stroke +TIA	74 (66–84)	51.5	33.0	64 s and 128 s	Single-phase; ECG-gated	Within 24 h
Tomari et al. (6)	314	Prospective	Stroke +TIA + SMS	70 (56–79)	61.1	23.9	384 s	Two-phase; non-gated	Median interval: 2.8 h
Yan et al. (31)	74	Prospective	ESUS	62 ± 14	62.2	0.0	64 s	Two-phase; ECG-gated	Within 1 week after stroke
Zhang et al. (3)	303	Prospective	CE	73 (65–80)	52.5	66.7	64 s	Two-phase; ECG-gated	Within 1 week after stroke

a The stroke type was defined based on information that excluded results of CCTA.

b Age was expressed as mean ± SD or median (low quartile, high quartile).

c Stroke patients with risk factors for cardiac thrombus.

AF, atrial fibrillation; CCTA, cardiac computed tomography angiography; CE, cardioembolic stroke; ES, embolic stroke; ESUS, embolic stroke of undetermined source; NR, not reported; SMS, stroke-mimicking syndromes; TEE, transesophageal echocardiography; TIA, transient ischemic attacks.

Risk-of-bias assessment showed that 12, 11, and two studies had a low, medium and high risk of bias, respectively. Thirteen studies utilized single-phase CCTA to detect cardiac thrombi, which is considered less accurate than dual-phase CCTA (32). Other significant sources of bias included small sample sizes and a low number of patients with cardiac thrombi. Additionally, several studies lacked crucial patient information in their reports, such as the incidence of atrial fibrillation and age. Furthermore, seven studies had a response rate less than 90%. Supplementary Table S4 summarizes the details of the risk of bias assessment.

### 3.2 Prevalence of cardiac thrombus detected by CCTA

**Figure 2:**
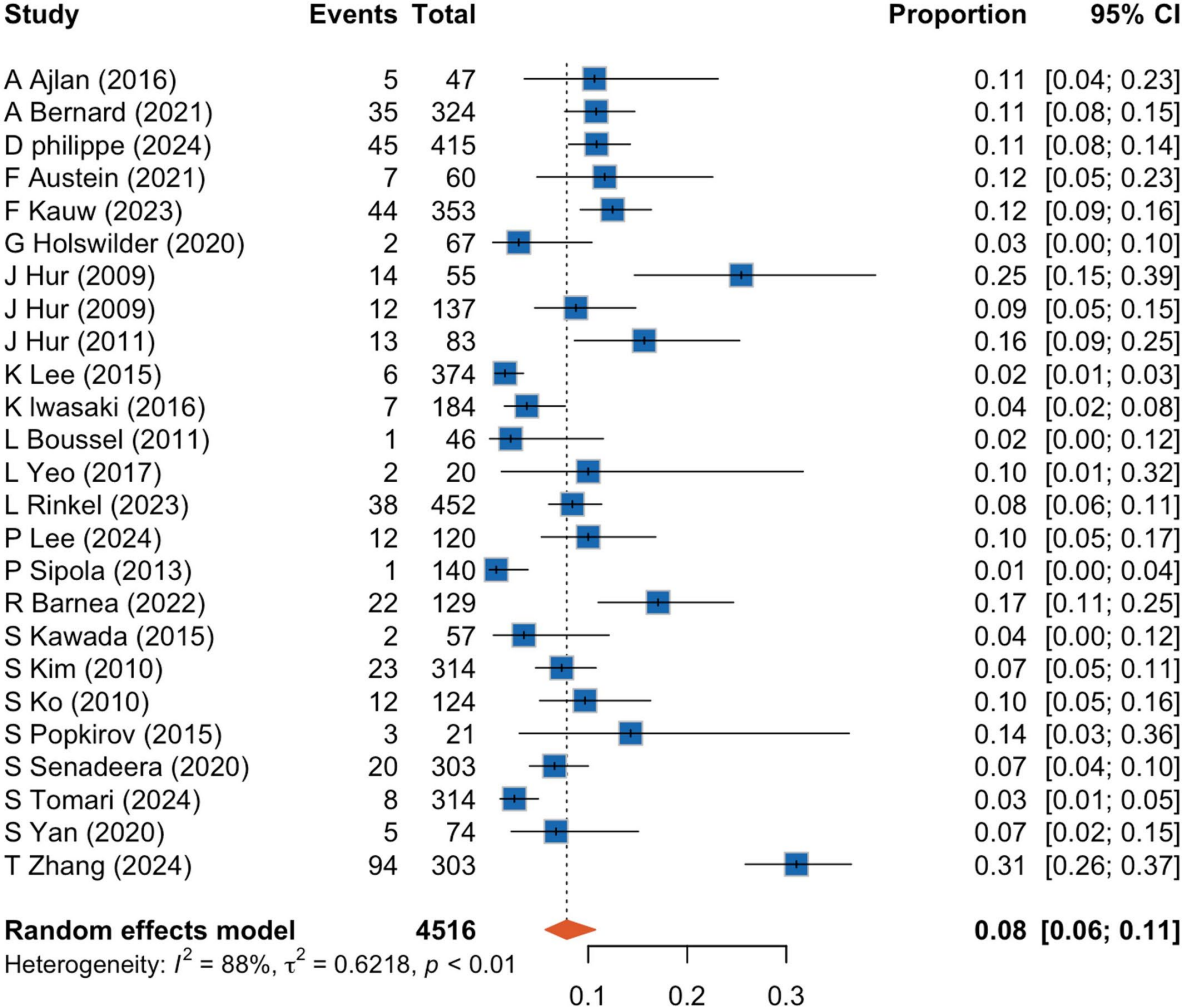
Forrest plot showing prevalence of cardiac thrombus detected by cardiac computed tomography angiography in acute ischemic stroke patients. CI, confidence interval.

**Figure 3:**
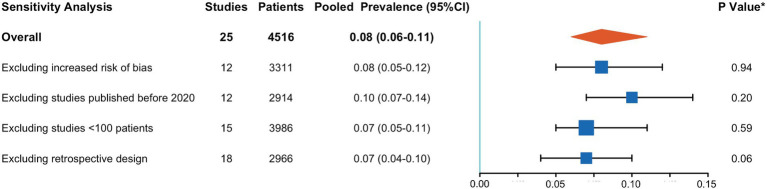
Results of sensitivity analysis. CI, confidence interval. *p-value for testing the null-hypothesis of homogeneity of cardiac thrombus prevalence between the included and excluded studies.

A meta-analysis of 25 studies indicated that CCTA identified intra-cardiac thrombi in 8% of patients with AIS (95% CI, 6–11%), with high interstudy heterogeneity (I^2^: 88%) (Figure 2). Sensitivity analyses (Figure 3) revealed that excluding studies with a medium or high risk of bias, studies conducted before 2020, studies with small sample sizes, or retrospective studies had no significant impact on the results (*p* > 0.05 for all comparisons). Furthermore, the removal of any individual trial did not substantially impact outcomes.

**Figure 4:**
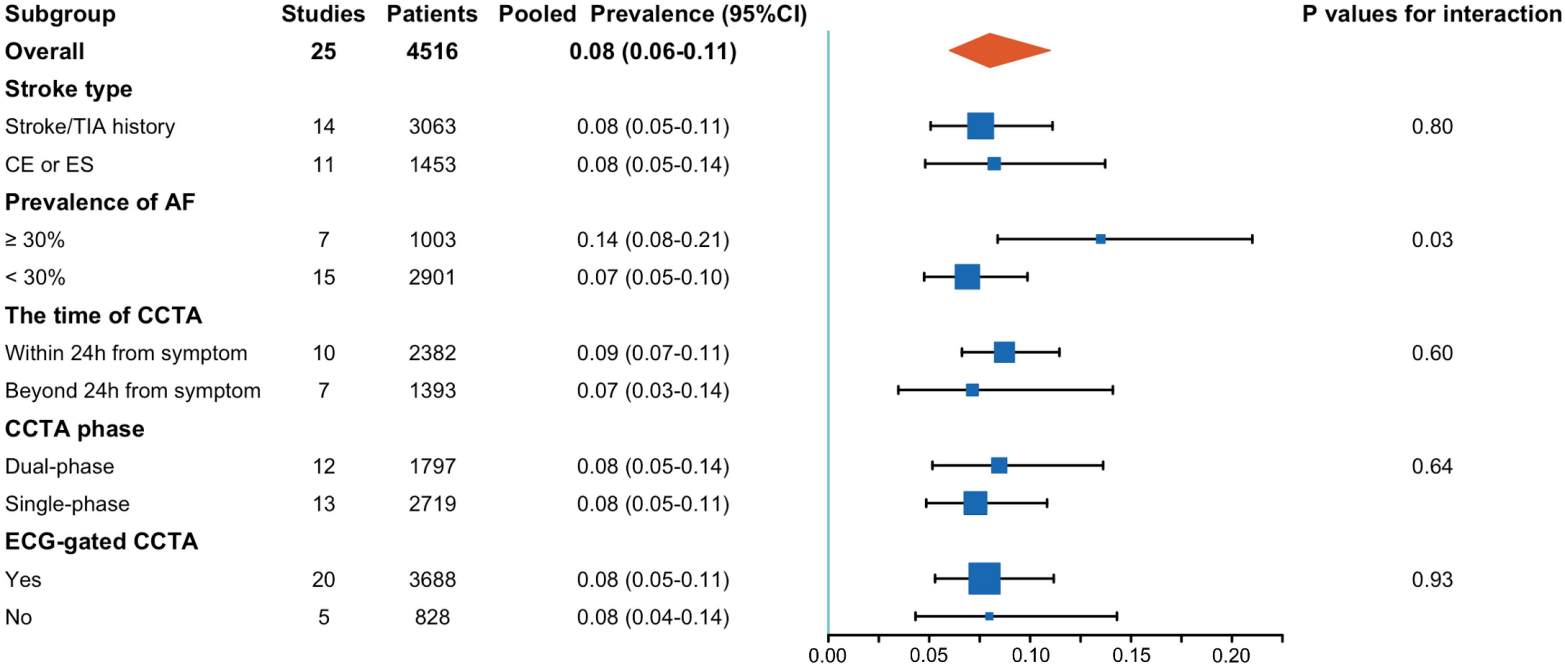
Results of subgroup analysis. AF, atrial fibrillation; CCTA, cardiac computed tomography angiography; CE, cardioembolic stroke; ES, embolic stroke.

Subgroup analyses revealed that a higher prevalence of atrial fibrillation significantly increased the detection rate of cardiac thrombi by CCTA (0.14; 95% CI, 0.08–0.21 vs. 0.07; 95% CI, 0.05–0.10; *p* = 0.03). Conversely, stroke type, interval from symptom onset to CCTA, and CCTA technology did not significantly influence the detection rate of cardiac thrombi. Details of these subgroup analyses are presented in Figure 4.

### 3.3 Atrial fibrillation and cardiac thrombus

Given that atrial fibrillation is a major contributor to cardioembolic stroke, we examined the prevalence of atrial fibrillation in patients with cardiac thrombi detected by CCTA. Fifteen studies reported the incidence of atrial fibrillation in patients with cardiac thrombus, encompassing a total of 318 patients. Notably, 41.5% (132/318) of the patients did not have documented atrial fibrillation (Table 2).

**Table 2:** Prevalence of atrial fibrillation in patients with cardiac thrombus and cardiac thrombus location.

Study	N	AF (N)	Location of cardiac thrombus		
			LAA/LA(N)	V(N)	RA(N)
Ajlan et al. (10)	5	NR	3	2	0
Bernard et al. (11)	35	22	35	0	0
Philippe et al. (12)	45	NR	41	3	1
Austein et al. (13)	7	NR	6	1	0
Kauw et al. (2)	44	19	35	14	0
Holswilder et al. (14)	2	NR	NR	NR	NR
Hur et al. (15)	14	10	14	0	0
Hur et al. (16)	12	NR	11	1	0
Hur et al. (17)	13	4	13	0	0
Lee et al. (18)	6	NR	NR	NR	NR
Iwasaki et al. (19)	7	3	6	1	0
Boussel et al. (20)	1	NR	0	1	0
Yeo et al. (21)	2	NR	1	1	0
Rinkel et al. (22)	38	15	33	5	0
Lee et al. (23)	12	NR	11	1	0
Sipola et al. (24)	1	0	0	1	0
Barnea et al. (25)	22	0	9	13	0
Kawada rt al. (26)	2	1	2	0	0
Kim et al. (27)	23	NR	23	0	0
Ko et al. (28)	12	11	11	1	0
Popkirov et al. (29)	3	3	2	0	1
Senadeera et al. (30)	20	15	20	0	0
Tomari et al. (6)	8	6	4	3	1
Yan et al. (31)	5	0	5	0	0
Zhang et al. (3)	94	77	NR	NR	NR

AF, atrial fibrillation; LA, left atrium; LAA, left atrial appendage; NR, not reported; RA, right atrium; V, ventricle.

### 3.4 Location of cardiac thrombus

Twenty-two studies provided data on the location of cardiac thrombi, revealing that 86.1% (285/331) of the thrombi were located in the LAA, followed by 14.5% (48/331) in the ventricles. Only three patients had thrombi in the right atrium (Table 2).

### 3.5 Publication bias

Visual assessment of the funnel plot (Supplementary Figure S1) did not indicate significant publication bias.

## 4 Discussion

To our knowledge, this is the first meta-analysis study to evaluate the prevalence of intracardiac thrombi detected by CCTA in patients with AIS. Our study suggests that cardiac thrombi can be detected on CCTA in approximately 8% of patients with AIS. Among stroke type, the prevalence of atrial fibrillation, timing of CCTA and CCTA technology, the prevalence of atrial fibrillation is the only factor associated with cardiac thrombi prevalence detected by CCTA. However, notably, almost half of the patients with cardiac thrombi detected by CCTA had no documented atrial fibrillation. The LAA and LA were the most common cardiac thrombus location, followed by the left ventricle.

Determining the etiology of a stroke is crucial for secondary prevention. Nevertheless, even after systematic evaluation, approximated one-third of strokes remain cryptogenic (1). In such cases, cardioembolic events may play a significant role, such as in patients with occult atrial fibrillation (1). Evaluating intracardiac thrombus during the acute phase of stroke may help identify the thrombus source and guide treatment decisions. Furthermore, current classification of cardioembolic stroke mainly relies on medical history and indirect examination results, such as the presence of atrial fibrillation, valvular heart disease, or ventricular aneurysm (1). In patients with stroke presenting risk factors for cardioembolism, atherosclerotic risk factors often co-exist, making the exact mechanism of stroke difficult to pinpoint. Additionally, the presence of an intracardiac thrombus is an important prognostic risk factor, associated with worse functional outcomes and longer hospital stay (4). In summary, acute phase assessment of intracardiac thrombus formation aids in the long-term secondary prevention for patients and help predict prognosis.

Nowadays, TEE is the gold-standard imaging modality for identifying intracardiac thrombi. When using intraoperative thrombus detection as the reference standard, TEE demonstrated a sensitivity of 93–100% and a specificity of 99–100% for detecting LAA thrombi. However, TEE is a semi-invasive and time-consuming procedure, and is not routinely performed in the acute period, potentially reducing the likelihood of detecting cardiac thrombus after intravenous thrombolysis and anti-thrombosis treatment. CCTA provides a non-invasive approach to detect cardiac thrombus and can be performed during the hyperacute period of AIS without significantly increasing additional scan time (22). A recent meta-analysis found that the prevalence of cardiac detected by CCTA was significantly higher than that detected by TEE in patients with AIS (33). However, the diagnostic accuracy of CCTA largely depends on the CT technology applied. Using TEE as the gold standard, dual-phase CCTA has shown high accuracy in diagnosing intracardiac thrombi, whereas single-phase CCTA is less accurate (32). Many studies on hyperacute CCTA in patients with AIS have adopted a single-phase scanning strategy to reduce scan time, potentially increasing the thrombus detection rate (2, 22). However, it is noteworthy that recent studies have found that filling defects (blood stasis) in single-phase CT scans, which might be misdiagnosed as thrombi, are also significantly associated with stroke recurrence, indicating that the etiology of stroke is probably cardiogenic thromboembolism (34). In addition, our study found that the timing of the scan had no significant impact on thrombus detection rates. Therefore, in the acute period of AIS, particularly in the hyperacute period, using single-phase scanning to reduce the scan time may be a reasonable choice.

To evaluate the proportion of cardiogenic thrombi detected by CCTA in patients with AIS, we pooled results from 25 studies and found that nearly 8% of patients with AIS had detectable thrombi on CCTA. The results showed considerable heterogeneity among the studies (Figure 2), which could be attributed to differences in study design, criteria for thrombus detection, patient characteristics, timing of CCTA, and technical variations in CCTA. To address this, we conducted sensitivity analyses and subgroup analyses based on study design and patient characteristics. However, following these analyses, the heterogeneity was not entirely explained by probable study biases. Thus, heterogeneity was likely attributed to patient-level factors and the heterogeneous nature of stroke etiology. Moreover, the included studies were not sufficiently high, with only about half being judged as high quality, which might induce heterogeneity among the studies. Interestingly, although it was speculated that factors such as stroke type, prevalence of atrial fibrillation among the study population, timing of CCTA, and CCTA scanning techniques could significantly influence thrombus detection rates, subgroup analysis revealed that the prevalence of atrial fibrillation among the study population was the sole risk factor affecting thrombus detection rates.

Among the 25 included studies, there was notable diversity in the types of strokes among the participants. Fourteen studies included all stroke types, including cases of TIA or stroke-mimicking symptoms. Conversely, some studies specifically focused on patients with cardioembolic or embolic stroke (Table 1). In the subgroup analysis, we did not observe a higher thrombus detection rate among cardioembolic or embolic stroke populations than general patients with stroke, and significant heterogeneity was evident within these groups. These confusing results likely stem from substantial inconsistencies in the clinical classification of stroke across different centers. It has been reported that the inter-center agreement regarding cardioembolic stroke was 38.7% (35). Furthermore, all included studies were observational single-center studies. There might be significant variations among the studies regarding the decision to perform CCTA in patients with AIS. In studies involving patients with unclassified stroke, those with cardioembolic stroke were still more likely to undergo CCTA examination, potentially introducing selection bias.

Among the enrolled 25 studies, only 10 studies conducted CCTA within 24 h of stroke onset, and only six studies provided precise timing details for CCTA. Our results did not support our hypothesis that earlier CCTA examinations would yield higher thrombus detection rates. Whether emergency CCTA scanning is warranted still requires further head-to-head comparison studies.

The prevalence of atrial fibrillation among the study participants was the sole risk factor associated with thrombus detection rates in our study. Although detecting cardioembolic thrombi may not significantly alter antithrombotic treatment decisions in patients with atrial fibrillation, thrombus detection can aid in prognosis prediction. Moreover, it is noteworthy that more than 40% of patients with detected thrombi did not have a history of atrial fibrillation. In these patients, thrombus detection holds significant implications for antithrombotic treatment decisions. This conclusion was recently validated by the ENCLOSE study, which demonstrated that CCTA-based thrombus detection significantly improved the diagnostic accuracy of cardioembolic stroke (2).

Finally, as expected, our study found that the LAA is a common site for thrombus formation; however, a significant proportion, approximately 14%, of thrombi can also be found in the ventricle. In rare cases, thrombi can occur in the right atrium. Due to their anatomical location away from the esophagus, thrombi in these areas can easily be missed during TEE, emphasizing the necessity of CCTA.

### 4.1 Limitation

This study had some limitations, including the great heterogeneity between studies, missing data, and low quality of the included studies, which might reduce the reliability of our results. Future high-quality prospective multicenter studies are warranted. Second, treatment decision changes and prognostic improvements resulting from CCTA findings are of great concern, however, few studies have reported on these outcomes. Future studies are needed to further establish the role of CCTA in improving the prognosis of AIS patients. Third, 11 studies focused on patients with embolic stroke, which might induce selection bias. However, in subgroup analysis, we did not demonstrate a significant impact of stroke type on the outcomes. Finally, various factors were associated with the incidence of intracardiac thrombus, such as age, heart failure, and left atrial size (36), which might influence the detection rate of cardiac thrombus on CCTA. However, due to missing data or a lack of significant differences among studies (e.g., age), we did not conduct subgroup analyses based on these factors.

## 5 Conclusion

CCTA might be a useful non-invasive imaging approach for evaluating cardiac embolism sources with cardiac thrombus being detected in approximately 8% of patients with AIS on CCTA. Atrial fibrillation was associated with an increased prevalence of cardiac thrombosis. However, the heterogeneity between studies necessitates future high-quality, large, multicenter prospective studies. Moreover, the optimal timing and technology aspects of CCTA require further studies.

## Data Availability

The original contributions presented in the study are included in the article/Supplementary material, further inquiries can be directed to the corresponding author.
