# Supplementary material for: Cardiac thrombus detected by cardiac computed tomography angiography in patients with acute ischemic stroke: a meta-analysis
Source: Front Neurol. 2024 Sep 10;15:1453683. doi: 10.3389/fneur.2024.1453683 (PMC11420050; doi:10.3389/fneur.2024.1453683)
Supplement: Supplementary file 1 [file Data_Sheet_1.docx]

**Supplementary Appendix**

**Supplementary Table 1.** PRISMA reporting checklist.

**Supplementary Table 2.** Search Strategy

**Supplementary Table 3.** Summary of the Joanna Briggs Institute Critical Appraisal Checklist for Studies Reporting Prevalence Data.

**Supplementary Table 4.** Risk of bias in included studies.

**Supplementary Figure 1.** Funnel plot of study size against logit-transformed proportion for all included studies.

**Supplemental Table 1.** PRISMA reporting checklist*. **Supplemental Table 1.** PRISMA reporting checklist*.

| **Section/topic** | **#** | **Checklist item** | **Reported on section** |
| --- | --- | --- | --- |
| **TITLE** | | | |
| Title | 1 | Identify the report as a systematic review, meta-analysis, or both. | Title |
| **ABSTRACT** | | | |
| Structured summary | 2 | Provide a structured summary including, as applicable: background; objectives; data sources; study eligibility criteria, participants, and interventions; study appraisal and synthesis methods; results; limitations; conclusions and implications of key findings; systematic review registration number. | Abstract |
| **INTRODUCTION** | | | |
| Rationale | 3 | Describe the rationale for the review in the context of what is already known. | Introduction |
| Objectives | 4 | Provide an explicit statement of questions being addressed with reference to participants, interventions, comparisons, outcomes, and study design (PICOS). | Introduction |
| **METHODS** | | | |
| Protocol and registration | 5 | Indicate if a review protocol exists, if and where it can be accessed (e.g., Web address), and, if available, provide registration information including registration number. | NR |
| Eligibility criteria | 6 | Specify study characteristics (e.g., PICOS, length of follow-up) and report characteristics (e.g., years considered, language, publication status) used as criteria for eligibility, giving rationale. | Methods |
| Information sources | 7 | Describe all information sources (e.g., databases with dates of coverage, contact with study authors to identify additional studies) in the search and date last searched. | Methods |
| Search | 8 | Present full electronic search strategy for at least one database, including any limits used, such that it could be repeated. | S.A. |
| Study selection | 9 | State the process for selecting studies (i.e., screening, eligibility, included in systematic review, and, if applicable, included in the meta-analysis). | Methods |
| Data collection process | 10 | Describe method of data extraction from reports (e.g., piloted forms, independently, in duplicate) and any processes for obtaining and confirming data from investigators. | Methods |
| Data items | 11 | List and define all variables for which data were sought (e.g., PICOS, funding sources) and any assumptions and simplifications made. | Methods |
| Risk of bias in individual studies | 12 | Describe methods used for assessing risk of bias of individual studies (including specification of whether this was done at the study or outcome level), and how this information is to be used in any data synthesis. | Methods and S.A. |
| Summary measures | 13 | State the principal summary measures (e.g., risk ratio, difference in means). | Methods |
| Synthesis of results | 14 | Describe the methods of handling data and combining results of studies, if done, including measures of consistency (e.g., I^2^) for each meta-analysis. | Methods |
| Risk of bias across studies | 15 | Specify any assessment of risk of bias that may affect the cumulative evidence (e.g., publication bias, selective reporting within studies). | Methods |
| Additional analyses | 16 | Describe methods of additional analyses (e.g., sensitivity or subgroup analyses, meta-regression), if done, indicating which were pre-specified. | Methods |
| **RESULTS** | | | |
| Study selection | 17 | Give numbers of studies screened, assessed for eligibility, and included in the review, with reasons for exclusions at each stage, ideally with a flow diagram. | Results, Figure 1 |
| Study characteristics | 18 | For each study, present characteristics for which data were extracted (e.g., study size, PICOS, follow-up period) and provide the citations. | Table 1 |
| Risk of bias within studies | 19 | Present data on risk of bias of each study and, if available, any outcome level assessment (see item 12). | Results, S.A. |
| Results of individual studies | 20 | For all outcomes considered (benefit or harms), present, for each study: (a) simple summary data for each intervention group (b) effect estimates and confidence intervals, ideally with a forest plot. | Results, Figure 2 |
| Synthesis of results | 21 | Present results of each meta-analysis done, including confidence intervals and measures of consistency. | Results, Figures 2 |
| Risk of bias across studies | 22 | Present results of any assessment of risk of bias across studies (see Item 15). | Results, S.A. |
| Additional analysis | 23 | Give results of additional analyses, if done (e.g., sensitivity or subgroup analyses, meta-regression [see Item 16]). | Results, Figures 3 – 4 |
| **DISCUSSION** | | | |
| Summary of evidence | 24 | Summarize the main findings including the strength of evidence for each main outcome; consider their relevance to key groups (e.g., healthcare providers, users, and policy makers). | Discussion |
| Limitations | 25 | Discuss limitations at study and outcome level (e.g., risk of bias), and at review-level (e.g., incomplete retrieval of identified research, reporting bias). | Limitations |
| Conclusions | 26 | Provide a general interpretation of the results in the context of other evidence, and implications for future research. | Conclusions |
| **FUNDING** | | | |
| Funding | 27 | Describe sources of funding for the systematic review and other support (e.g., supply of data); role of funders for the systematic review. | Funding |

* From: Moher D, Liberati A, Tetzlaff J, Altman DG, for the PRISMA Group. Preferred reporting items for systematic reviews and meta-analyses: the PRISMA statement. BMJ 2009;339:b2535–b2535.

N.R.: not reported, PRISMA: preferred reporting items for systematic reviews and meta-analyses, S.A.: supplemental appendix.

| **Supplemental Table 2. Search Strategy** | |
| --- | --- |
| Embase | N |
| #1: ‘cerebrovascular disease’/de OR ‘basal ganglia infarction’/exp OR ‘brain ischemia’/exp OR ‘ischemic stroke’/exp OR ‘acute ischemic stroke’/de OR ‘brain infarction’/exp OR ‘carotid artery disease’/exp OR ‘cerebral artery disease’/de OR ‘anterior circulation infarction’/exp OR ‘posterior circulation infarction’/de OR ‘brain embolism’/de OR ‘cerebrovascular accident’/exp | 770343 |
| #2: ‘isch$emi*’ NEAR/5 (‘stroke*’ OR ‘apoplex*’ OR ‘cerebral vasc*’ OR ‘cerebrovasc*’ OR ‘cva’ OR ‘attack*’) | 205156 |
| #3: (‘brain’ OR ‘cerebr*’ OR ‘cerebell*’ OR ‘vertebrobasil*’ OR ‘hemispher*’ OR ‘intracran*’ OR ‘intracerebral’ OR ‘infratentorial’ OR ‘supratentorial’ OR ‘middle cerebr*’ OR ‘mca’ OR ‘anterior circulation’ OR ‘basilar artery’ OR ‘vertebral artery’ OR ‘basal gangli*’) NEAR/5 (‘isch$emi*’ OR ‘infarct*’ OR ‘thrombo*’ OR ‘emboli*’ OR ‘occlus*’ OR ‘hypoxi*’) | 343339 |
| #4: #1 or #2 or #3 | 846461 |
| #5: (‘appendage’ OR ‘atrial’ OR ‘ventricle’ or ‘cardiac’ or ‘heart’) adj5 (‘compute* tomograph*’ or ‘compute* aided tomograph*’ or ‘CT’ or ‘CCT’). ti,ab,kw. or ((‘heart’/exp or ‘heart atrium’/exp or ‘heart ventricle’/exp) and ‘computed tomographic angiography’/exp) | 13512 |
| #6: #4 and #5 | 1345 |
| Web of Science |  |
| #1: (‘isch$emi*’ NEAR/5 (‘stroke*’ OR ‘apoplex*’ OR ‘cerebral vasc*’ OR ‘cerebrovasc*’ OR ‘cva’ OR ‘attack*’)) | 222973 |
| #2: ((‘brain’ OR ‘cerebr*’ OR ‘cerebell*’ OR ‘vertebrobasil*’ OR ‘hemispher*’ OR ‘intracran*’ OR ‘intracerebral’ OR ‘infratentorial’ OR ‘supratentorial’ OR ‘middle cerebr*’ OR ‘mca’ OR ‘anterior circulation’ OR ‘basilar artery’ OR ‘vertebral artery’ OR ‘basal gangli*’) NEAR/5 (‘isch$emi*’ OR ‘infarct*’ OR ‘thrombo*’ OR ‘emboli*’ OR ‘occlus*’ OR ‘hypoxi*’)) | 370336 |
| #3: #1 or #2 | 467325 |
| #4: ((‘appendage’ OR ‘atrial’ OR ‘ventricle’ OR ‘cardiac’ OR ‘heart’) NEAR/5 (‘computed tomography’ OR ‘computed aided tomography’ OR ‘CT’ OR ‘CCT’)) or ((‘heart’ OR ‘heart atrium’ OR ‘heart ventricle’) AND ‘computed tomographic angiography’) | 25690 |
| #5: #3 and #4 | 874 |
| MEDLINE |  |
| #1: cerebrovascular disorders/ OR brain ischemia/ OR brain infarction/ OR brain stem infarctions/ OR cerebral infarction/ OR infarction, anterior cerebral artery/ OR infarction, middle cerebral artery/ OR infarction, posterior cerebral artery/ OR ischemic attack, transient/ OR intracranial embolism and thrombosis/ OR intracranial embolism/ OR intracranial thrombosis/ OR stroke/ OR exp ischemic stroke/ | 51043 |
| #2: TX (stroke OR poststroke OR post‐stroke OR cerebrovasc$ OR (cerebr$ adj3 vasc$) OR CVA$ OR apoplectic OR apoplex$ OR (transient N3 isch?emic N3 attack) OR tia$)) | 76369 |
| #3: ((TX (cerebr* OR cerebell* OR arteriovenous OR vertebrobasil* OR interhemispheric OR hemispher* OR intracran* OR intracerebral OR infratentorial OR supratentorial OR MCA* OR (anterior N3 circulat*) OR (posterior N3 circulat*) OR lenticulostriate OR (middle N3 arter*) OR (basilar N3 arter*) OR (brachial N3 arter*) OR (vertebr* N3 arter*))) AND (TX ((blood N5 clot*) OR disease* OR damage* OR disorder* OR disturbance OR dissection OR lesion OR syndrome OR arrest OR accident OR lesion OR vasculopathy OR insult OR attack OR injury OR insufficiency OR malformation OR obstruct* OR anomal*))) | 81540 |
| #4: (TX (cerebr* OR cerebell* OR arteriovenous OR vertebrobasil* OR interhemispheric OR hemispher* OR intracran* OR "corpus callosum" OR intracerebral OR intracortical OR intraventricular OR periventricular OR "posterior fossa" OR infratentorial OR supratentorial OR MCA* OR (anterior N3 circulation) OR (posterior N3 circulation) OR "basal ganglia" OR (middle N3 arter*) OR (basilar N3 arter*) OR (brachial N3 arter*) OR (vertebr* N3 arter*) OR "space-occupying" OR "brain ventricle*" OR lacunar OR cortical OR ocular)) AND (TX (isch?emi* OR infarct* OR thrombo* OR emboli* OR occlus* OR hypoxi* OR vasospasm OR obstruct* OR vasoconstrict*)) | 26996 |
| #5: #1 or #2 or #3 or #4 | 144699 |
| #6: ((TI (appendage OR atrial OR ventricle OR cardiac OR heart) N5 (computed tomography OR computed aided tomography OR CT OR CCT)) OR (AB (appendage OR atrial OR ventricle OR cardiac OR heart) N5 (computed tomography OR computed aided tomography OR CT OR CCT)) OR (KW (appendage OR atrial OR ventricle OR cardiac OR heart) N5 (computed tomography OR computed aided tomography OR CT OR CCT))) OR ((MH ‘Heart’ OR MH ‘Heart Atria’ OR MH ‘Heart Ventricles’) AND MH ‘Computed Tomography Angiography’) | 12863 |
| #7: #5 and #6 | 744 |
| CENTRAL |  |
| #1: ‘MeSH descriptor: [Cerebrovascular Disorders] this term only’ OR ‘MeSH descriptor: [Basal Ganglia Cerebrovascular Disease] this term only’ OR ‘MeSH descriptor: [Brain Ischemia] explode all trees’ OR ‘MeSH descriptor: [Carotid Artery Diseases] this term only’ OR ‘MeSH descriptor: [Carotid Artery Thrombosis] this term only’ OR ‘MeSH descriptor: [Intracranial Arterial Diseases] this term only’ OR ‘MeSH descriptor: [Cerebral Arterial Diseases] this term only’ OR ‘MeSH descriptor: [Intracranial Embolism and Thrombosis] explode all trees’ OR ‘MeSH descriptor: [Stroke] explode all trees’ OR ((isch?emi* NEAR/6 (stroke* OR apoplex* OR cerebral vasc* OR cerebrovasc* OR cva OR attack*)):ti,ab,kw) OR (((brain OR cerebr* OR cerebell* OR vertebrobasil* OR hemispher* OR intracran* OR intracerebral OR infratentorial OR supratentorial OR middle cerebr* OR mca* OR anterior circulation) NEAR/5 (isch?emi* OR infarct* OR thrombo* OR emboli* OR occlus* OR hypoxi*)):ti) | 31122 |
| #2: (((appendage OR atrial OR ventricle OR cardiac OR heart) NEAR/5 (computed tomography OR computed aided tomography OR CT OR CCT))) OR ((MeSH descriptor: [Computed Tomography Angiography] explode all trees) AND (MeSH descriptor: [Heart] explode all trees)) | 1093 |
| #3: #1 and #2 | 137 |

| **Supplemental Table 3.** Summary of the Joanna Briggs Institute Critical Appraisal Checklist for Studies Reporting Prevalence Data* | |
| --- | --- |
| **Questions** | **Details** |
| 1. Was the sample frame appropriate to address the target population? | Assessed each study’s patient characteristics and whether these patients are reflective of our intended study population. |
| 2. Were study participants sampled in an appropriate way? | Assessed how each study sampled and selected their patients. |
| 3. Was the sample size adequate? | Assessed whether the study size of each included study was appropriate for detecting cardiac thrombus by cardiac CT angiography (Sample size >100). |
| 4. Were the study subjects and the setting described in detail? | Assessed each study’s description of patients, definitions and interventions. |
| 5. Was the data analysis conducted with sufficient coverage of the identified sample? | Assessed the response rate of study patients. |
| 6. Were valid methods used for the identification of the condition? | Assessed the methods used to diagnose cardiac thrombus (whether dual-phase CT scan was conducted to diagnose cardiac thrombus or not). |
| 7. Was the condition measured in a standard, reliable way for all participants? | Assessed how each study diagnosed cardiac thrombus. |
| 8. Was there appropriate statistical analysis? | Assessed study numerical and statistical reporting. |
| 9. Was the response rate adequate, and if not, was the low response rate managed appropriately? | Assessed the drop-out rate of patients in each study (less than 10%). |

* From: Munn Z, Moola S, Lisy K, Riitano D, Tufanaru C. Methodological guidance for systematic reviews of observational epidemiological studies reporting prevalence and cumulative incidence data: Int J of Evid Based Healthc. 2015; 13:147–153.

| **Supplemental Table 4.** Risk of bias in included studies* | | | | | | | | | | | |
| --- | --- | --- | --- | --- | --- | --- | --- | --- | --- | --- | --- |
| Study | Was the sample frame appropriate to address the target population? | Were study participants sampled in an appropriate way? | Was the sample size adequate? | Were the study subjects and the setting described in detail? | Was the data analysis conducted with sufficient coverage of the identified sample? | Were valid methods used for the identification of the condition? | Was the condition measured in a standard reliable way for all participants? | Was there appropriate statistical analysis? | Was the response rate adequate, and if not, was the low response rate managed appropriately? | Score† | Risk of Bias‡ |
| A Ajlan (2016) | Yes | Yes | No | Yes | Yes | No | Yes | Yes | Yes | 7 | Medium |
| A Bernard (2021) | Yes | Yes | Yes | Yes | Yes | No | Yes | Yes | Yes | 8 | Low |
| D Philippe (2024) | Yes | Yes | Yes | No | Yes | No | Yes | Yes | Yes | 7 | Medium |
| F Austein (2021) | Yes | Yes | No | Yes | Yes | Yes | Yes | Yes | No | 7 | Medium |
| F Kauw (2023) | Yes | Yes | Yes | Yes | Yes | No | Yes | Yes | Yes | 8 | Low |
| G Holswilder (2020) | Yes | Yes | No | Yes | No | No | Yes | Yes | No | 5 | High |
| J Hur (2009) | Yes | Yes | No | No | No | Yes | Yes | Yes | Yes | 6 | Medium |
| J Hur (2009) | Yes | Yes | Yes | Yes | Yes | Yes | Yes | Yes | Yes | 9 | Low |
| J Hur (2011) | Yes | Yes | No | Yes | Yes | No | Yes | Yes | No | 6 | Medium |
| K Lee (2015) | Yes | Yes | Yes | Yes | Yes | No | Yes | Yes | Yes | 8 | Low |
| K lwasaki (2016) | Yes | Yes | Yes | Yes | Yes | Yes | Yes | Yes | Yes | 9 | Low |
| L Boussel (2011) | Yes | Yes | No | Yes | No | Yes | Yes | Yes | Yes | 7 | Medium |
| L Yeo (2017) | Yes | Yes | No | Yes | No | No | Yes | Yes | Yes | 6 | Medium |
| L Rinkel (2023) | Yes | Yes | Yes | Yes | Yes | No | Yes | Yes | Yes | 8 | Low |
| P Lee (2024) | Yes | Yes | No | Yes | No | No | Yes | Yes | Yes | 6 | Medium |
| P Sipola (2013) | Yes | Yes | Yes | No | No | No | Yes | Yes | Yes | 6 | Medium |
| R Barnea (2022) | Yes | Yes | Yes | Yes | Yes | Yes | Yes | Yes | No | 8 | Low |
| S Kawada (2015) | Yes | Yes | No | No | No | Yes | Yes | Yes | No | 5 | High |
| S Kim (2010) | Yes | Yes | Yes | Yes | Yes | Yes | Yes | Yes | Yes | 9 | Low |
| S Ko (2010) | Yes | Yes | Yes | Yes | Yes | Yes | Yes | Yes | Yes | 9 | Low |
| S Popkirov (2015) | Yes | Yes | Yes | Yes | Yes | No | Yes | Yes | No | 7 | Medium |
| S Senadeera (2020) | Yes | Yes | Yes | Yes | Yes | No | Yes | Yes | Yes | 8 | Low |
| S Tomari (2024) | Yes | Yes | Yes | Yes | Yes | Yes | Yes | Yes | Yes | 9 | Low |
| S Yan (2020) | Yes | Yes | No | Yes | Yes | Yes | Yes | Yes | No | 7 | Medium |
| T Zhang (2024) | Yes | Yes | Yes | Yes | Yes | Yes | Yes | Yes | Yes | 9 | Low |

* From: Munn Z, Moola S, Lisy K, Riitano D, Tufanaru C. Methodological guidance for systematic reviews of observational epidemiological studies reporting prevalence and cumulative incidence data: Int J of Evid Based Healthc. 2015; 13:147–153.

†A summated score for each question item result. For each question item, “Unclear” and “No” score 0, “Yes” scores 1.

‡ Derived from the summated score with a low risk of bias scoring 8-9, a medium risk of bias scoring 6-7, and a high risk of bias scoring 0-5.


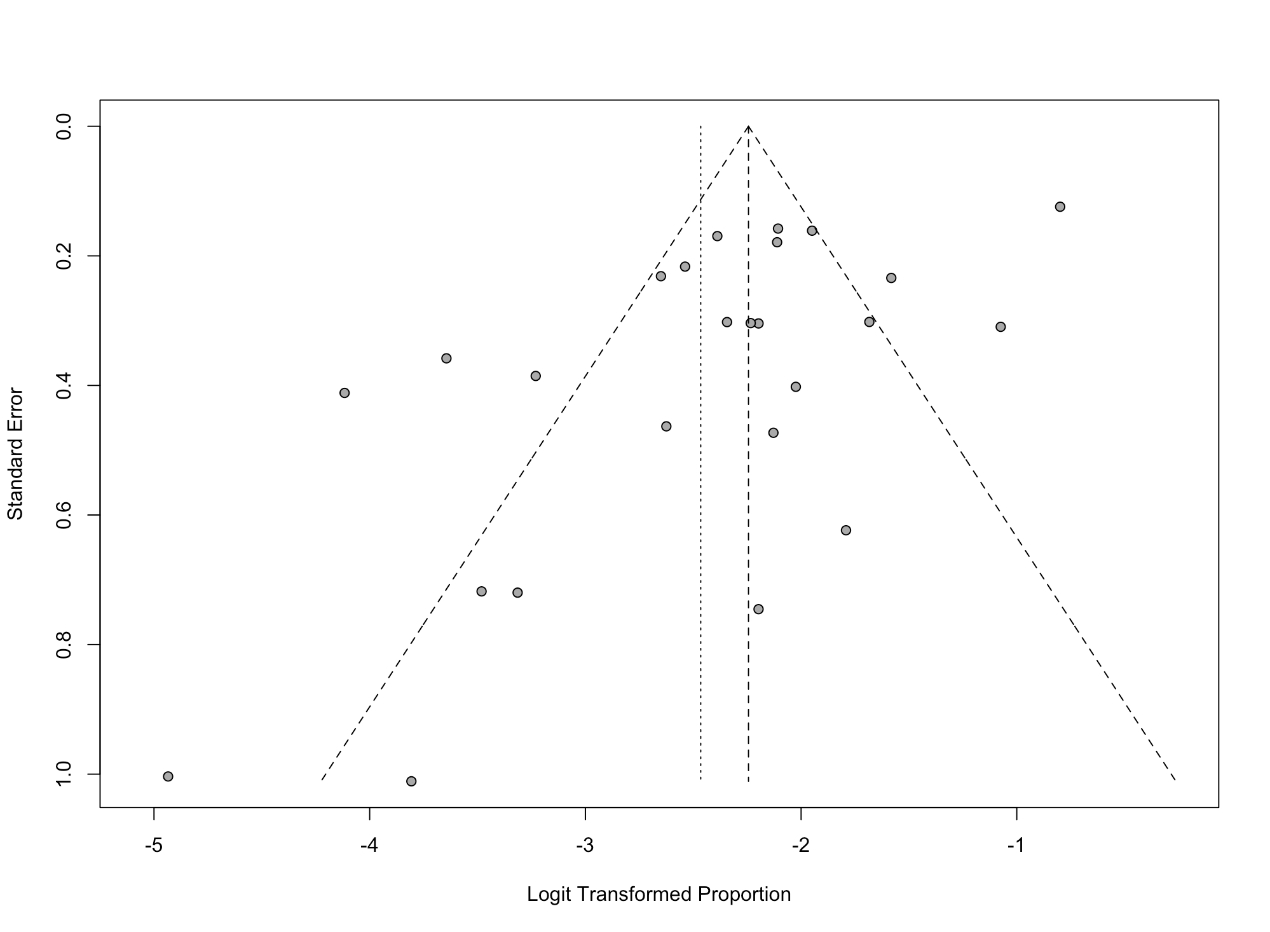


**Supplemental Figure 1.** Funnel plot of study size against logit-transformed proportion for all included studies.
